# Drugs Associated With the Development of Palmoplantar Keratoderma: A Systematic Review

**DOI:** 10.1177/12034754211004560

**Published:** 2021-03-28

**Authors:** Sara Mirali, Abrahim Abduelmula, Asfandyar Mufti, Muskaan Sachdeva, Jensen Yeung

**Affiliations:** 1 12366 Faculty of Medicine, University of Toronto, Canada; 2 70384 Faculty of Medicine, University of Western Ontario, London, Canada; 3 12366 Division of Dermatology, Department of Medicine, University of Toronto, Canada; 4 Sunnybrook Health Sciences Centre, Toronto, ON, Canada; 5 Women’s College Hospital, Toronto, ON, Canada; 6 Probity Medical Research Inc., Waterloo, ON, Canada

**Keywords:** palmoplantar keratoderma, adverse drug reactions, BRAF inhibitors, tyrosine kinase inhibitors

## Abstract

**Background:**

Palmoplantar keratoderma (PPK) are a heterogenous group of hereditary and acquired disorders that are characterized by excessive epidermal thickening of the palms and/or soles. PPK has been described as a rare adverse event for some medications. The aim of this systematic review was to summarize outcomes in PPK associated with various medications. This data will assist dermatologists and other healthcare providers treating patients with drug-induced PPK.

**Methods:**

EMBASE and MEDLINE databases were searched in accordance with PRISMA guidelines using the keyword “palmoplantar keratoderma.” 40 studies met the inclusion criteria.

**Results:**

A total of 247 patients (mean age: 57.0 years) were included in the analysis. Among patients whose sex was reported, 60.3% (*n* = 35/58) were male. PPK most frequently developed after treatment with BRAF inhibitors (73.7%, *n* = 182/247), BRAF inhibitors combined with MEK1/2 inhibitors (15.4%, *n* = 38/247), tyrosine kinase inhibitors (TKIs) (3.2%, *n* = 8/247), or chemotherapy (2.4%, *n* = 6/247). The mean latency period between initiation of the drug and onset of PPK was 7.6 months (range: 0.25-90 months). Improvement of PPK was reported in 24 cases, with 50% (*n* = 12/24) achieving complete resolution and 50% (*n* = 12/24) achieving partial resolution. All patients who achieved complete resolution stopped the suspected drug, with a mean resolution period of 2.4 months (range: 2 weeks-6 months). The most common treatments for PPK were keratolytic treatments (*n* = 10) and topical corticosteroids (*n* = 4).

**Conclusions:**

PPK was most frequently associated with targeted kinase inhibitors, specifically BRAF, MEK1/2, and tyrosine kinase inhibitors.

Palmoplantar keratoderma (PPK) are a heterogenous group of hereditary and acquired disorders that are characterized by excessive epidermal thickening of the palms and/or soles. PPK has recently been described as a rare cutaneous complication of some medications and can significantly impact patients’ quality of life. The aim of this systematic review was to summarize outcomes in PPK associated with various medications.

EMBASE and MEDLINE databases were searched on October 21, 2020 in accordance with PRISMA guidelines using the keyword “palmoplantar keratoderma” (Supplemental Figures S1-S2, Supplemental Table S1). Of the 40 studies (23 case reports, 9 case series, 6 cohort studies, and 2 randomized controlled trial) that met the inclusion criteria, a total of 247 patients (mean age: 57.0 years) were included. Among patients whose sex was reported, 60.3% (*n* = 35/58) were male (Supplemental Table S2). PPK most frequently developed after treatment with BRAF inhibitors (73.7%, n = 182/247), BRAF inhibitors combined with MEK1/2 inhibitors (15.4%, *n* = 38/247), tyrosine kinase inhibitors (3.2%, *n* = 8/247), or chemotherapy (2.4%, *n* = 6/247).

The mean latency period between initiation of the drug and onset of PPK was 7.6 months (range: 0.25-90 months) (Supplemental Table S3). Of the 24 cases that reported improvement of PPK, 50.0% (*n* = 12/24) had complete and 50.0% (*n* = 12/24) had partial resolution. All patients who achieved complete resolution stopped the suspected drug and the mean resolution period was 2.4 months. Among those with partial resolution, the drug was discontinued in 41.7% (*n* = 5/12) of cases and continued in 25.0% (*n* = 3/12). One patient who did not discontinue the drug was treated with systemic corticosteroids, achieving partial resolution in 4 months. The overall mean resolution period for patients achieving partial resolution was 3.0 months. Treatments for PPK included keratolytic treatments (*n* = 10), topical corticosteroids (*n* = 4), systemic corticosteroids (n = 4), antihistamines (*n* = 2), and retinoids (*n* = 2).

PPK was most frequently associated with BRAF inhibitors, which are used to target malignancies harboring *BRAF* mutations. Pharmacological inhibition of BRAF signaling in normal skin cells increases MAPK/ERK (mitogen-activated protein kinase/extracellular signal-regulated kinase) signaling through CRAF, resulting in increased keratinocyte proliferation.^1^ To reduce the risk of PPK and other proliferative cutaneous side effects, BRAF inhibitors can be paired with MEK1/2 inhibitors to block MAPK/ERK signaling downstream. Several studies showed a reduction in PPK occurrence when BRAF and MEK1/2 inhibitors were used in combination.^2-4^ PPK also occurred after treatment with chemotherapy, such as capecitabine. While the pathogenesis is unknown, one theory suggests that capecitabine is eliminated by the eccrine system, resulting in off-target toxicity in the palm and soles.^5^ However, further studies need to investigate why keratinocytes are unaffected by capecitabine’s main metabolite, 5-fluorouracil, which blocks DNA synthesis and cell proliferation.

Our systematic review has some limitations. The majority of the included studies were case reports or case series, which limits the generalizability of our analysis. In addition, the mean Naranjo score was 5, which suggests a “probable” association between initiation of the suspected drug and development of PPK. Despite these limitations, our review provides important information about the occurrence of PPK as an adverse drug reaction, most commonly noted with BRAF inhibitors.

## Supplemental Material

Supplementary material - Supplemental material for Drugs Associated With the Development of Palmoplantar Keratoderma: A Systematic Review Click here for additional data file. Supplemental material, Supplementary material, for Drugs Associated With the Development of Palmoplantar Keratoderma: A Systematic Review by Sara Mirali, Abrahim Abduelmula, Asfandyar Mufti, Muskaan Sachdeva and Jensen Yeung in Journal of Cutaneous Medicine and Surgery
