# Supplementary material for: Drugs Associated With the Development of Palmoplantar Keratoderma: A Systematic Review
Source: J Cutan Med Surg. 2021 Mar 28;25(5):553–4. doi: 10.1177/12034754211004560 (PMC8474319; doi:10.1177/12034754211004560)
Supplement: Supplementary material - Supplemental material for Drugs Associated With the Development of Palmoplantar Keratoderma: A Systematic Review [file sj-pdf-1-cms-10.1177_12034754211004560.pdf]

Supplemental Material

Drugs Associated with the Development of Palmoplantar Keratoderma: A Systematic Review

Sara Mirali (PhD)[1], Abrahim Abduelmula (BScN)[2], Asfandyar Mufti (MD)[3], Muskaan Sachdeva (BHSc)[1], Jensen Yeung (MD, FRCPC)[3,4,5,6]

[1]Faculty of Medicine, University of Toronto, Canada

[2]Faculty of Medicine, University of Western Ontario, London, Canada

[3]Division of Dermatology, Department of Medicine, University of Toronto, Toronto, Canada

[4]Sunnybrook Health Sciences Centre, Toronto, ON, Canada

[5]Women's College Hospital, Toronto, ON, Canada

[6]Probity Medical Research Inc., Waterloo, ON, Canada

Corresponding author:

Jensen Yeung, Women's College Hospital, Division of Dermatology.

76 Greenville St, 5th floor, Toronto, ON M5S 1B2, Canada.

Email: jensen.yeung@utoronto.ca

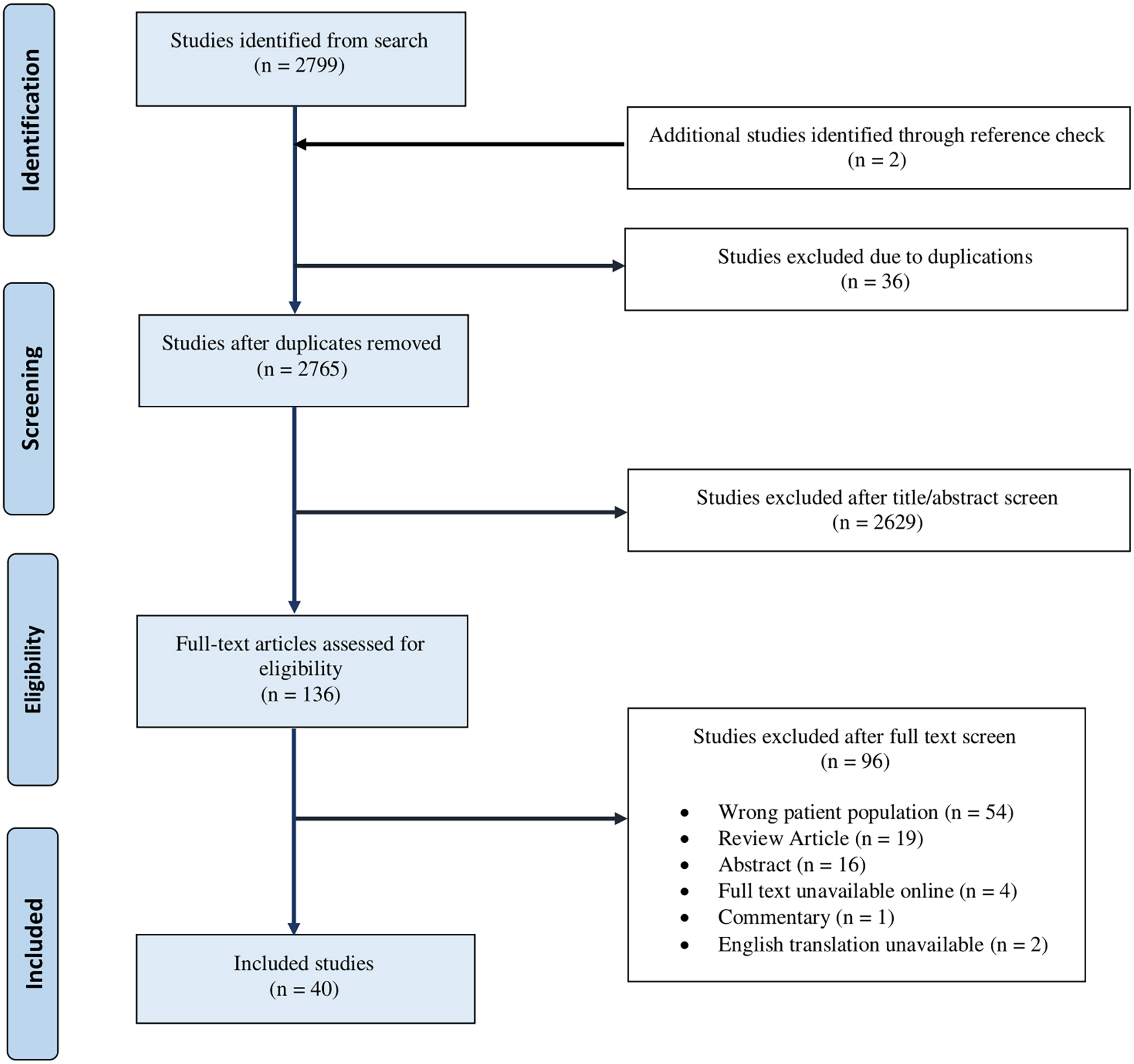

Figure S1: Literature screening flow diagram using the Preferred Reporting Items for Systematic Reviews and Meta-Analyses (PRISMA) guidelines. Figure adapted from http://prisma-statement.org.

PRISMA 2009 Checklist

Section/topic	#	Checklist item	Reported on page #
**TITLE**			1
Title	1	Identify the report as a systematic review, meta-analysis, or both.	Yes
**ABSTRACT**			Not needed for this article type
Structured summary	2	Provide a structured summary including, as applicable: background; objectives; data sources; study eligibility criteria, participants, and interventions; study appraisal and synthesis methods; results; limitations; conclusions and implications of key findings; systematic review registration number.	N/A
**INTRODUCTION**			2
Rationale	3	Describe the rationale for the review in the context of what is already known.	Yes
Objectives	4	Provide an explicit statement of questions being addressed with reference to participants, interventions, comparisons, outcomes, and study design (PICOS).	Yes
**METHODS**			2
Protocol and registration	5	Indicate if a review protocol exists, if and where it can be accessed (e.g., Web address), and, if available, provide registration information including registration number.	The protocol was not registered on Prospero.
Eligibility criteria	6	Specify study characteristics (e.g., PICOS, length of follow-up) and report characteristics (e.g., years considered, language, publication status) used as criteria for eligibility, giving rationale.	Yes
Information sources	7	Describe all information sources (e.g., databases with dates of coverage, contact with study authors to identify additional studies) in the search and date last searched.	Yes
Search	8	Present full electronic search strategy for at least one database, including any limits used, such that it could be repeated.	Yes
Study selection	9	State the process for selecting studies (i.e., screening, eligibility, included in systematic review, and, if applicable, included in the meta-analysis).	Yes
Data collection process	10	Describe method of data extraction from reports (e.g., piloted forms, independently, in duplicate) and any processes for obtaining and confirming data from investigators.	Yes
Data items	11	List and define all variables for which data were sought (e.g., PICOS, funding sources) and any assumptions and simplifications made.	Yes
Risk of bias in individual studies	12	Describe methods used for assessing risk of bias of individual studies (including specification of whether this was done at the study or outcome level), and how this information is to be used in any data synthesis.	Yes
Summary measures	13	State the principal summary measures (e.g., risk ratio, difference in means).	Yes

Synthesis of results	14	Describe the methods of handling data and combining results of studies, if done, including measures of consistency (e.g., I^2) for each meta-analysis.	N/A

Section/topic	#	Checklist item	Reported on page #
Risk of bias across studies	15	Specify any assessment of risk of bias that may affect the cumulative evidence (e.g., publication bias, selective reporting within studies).	Yes
Additional analyses	16	Describe methods of additional analyses (e.g., sensitivity or subgroup analyses, meta-regression), if done, indicating which were pre-specified.	N/A
**RESULTS**			2
Study selection	17	Give numbers of studies screened, assessed for eligibility, and included in the review, with reasons for exclusions at each stage, ideally with a flow diagram.	Yes
Study characteristics	18	For each study, present characteristics for which data were extracted (e.g., study size, PICOS, follow-up period) and provide the citations.	Yes
Risk of bias within studies	19	Present data on risk of bias of each study and, if available, any outcome level assessment (see item 12).	Yes
Results of individual studies	20	For all outcomes considered (benefits or harms), present, for each study: (a) simple summary data for each intervention group (b) effect estimates and confidence intervals, ideally with a forest plot.	Yes (a only)
Synthesis of results	21	Present results of each meta-analysis done, including confidence intervals and measures of consistency.	N/A
Risk of bias across studies	22	Present results of any assessment of risk of bias across studies (see Item 15).	Yes
Additional analysis	23	Give results of additional analyses, if done (e.g., sensitivity or subgroup analyses, meta-regression [see Item 16]).	N/A
**DISCUSSION**			2-3
Summary of evidence	24	Summarize the main findings including the strength of evidence for each main outcome; consider their relevance to key groups (e.g., healthcare providers, users, and policy makers).	Yes
Limitations	25	Discuss limitations at study and outcome level (e.g., risk of bias), and at review-level (e.g., incomplete retrieval of identified research, reporting bias).	Yes
Conclusions	26	Provide a general interpretation of the results in the context of other evidence, and implications for future research.	Yes
**FUNDING**			N/A
Funding	27	Describe sources of funding for the systematic review and other support (e.g., supply of data); role of funders for the systematic review.	N/A

From: Moher D, Liberati A, Tetzlaff J, Altman DG, The PRISMA Group (2009). Preferred Reporting Items for Systematic Reviews and Meta-Analyses: The PRISMA Statement. PLoS Med 6(7): e1000097. doi:10.1371/journal.pmed1000097

For more information, visit: **www.prisma-statement.org**.

Table S1. Search Strategy

#	Searches	Results
1	palmoplantar keratoderma.mp. [mp=ti, ab, ot, nm, hw, fx, kf, ox, px, rx, ui, an, sy, tn, dm, mf, dv, kw, dq]	3822
2	remove duplicates from 1	2799

Table S2. Summary of Included Cases of Drug-Induced Palmoplantar Keratoderma

Case Reports and Case Series																			
Study Information		Patient Information				Drug Information				PPK Information			PPK Resolution						
**Study design (Level of Evidence)**	**Sample size (n/N)**	**Age, Sex**	**History of skin disease**	**Comorbidities**	**Indication**	**Previous treatments for indication**	**Drug (dose and frequency)**	**Drug class**	**Concurrent medications (dose and frequency)**	**PPK onset (Months)**	**Description of PPK**	**Histopathological findings**	**Drug discontinued? (Y/N)**	**Treatment for PPK (dose and frequency)**	**Medications prescribed for original indication**	**Resolution period (months)**	**Resolution (CoR, PR, NoR)**	**PPK Recurrence (Y/N)**	**Naranjo ADR scale**
CR (5)[1]	1	58, M	NR	NR	Langerhans cell histiocytosis	cyclophosphamide, doxorubicin, vincristine, and prednisone (NR,NR)	dabrafenib (150 mg,BID)	BRAF inhibitor	NR	3	hyperkeratosis	NR	Y	NR	NR	NR	NR	NR	5
CS (4)[2]	5/14	52, M	NR	NR	metastatic melanoma	NR	dabrafenib (NR,NR)	BRAF inhibitor	NR	1.75	diffuse yellowish hyperkeratosis	NR	NR	keratolytic creams (NR,NR)	NR	NR	NR	NR	4
		65, M	NR	NR	metastatic melanoma	NR	dabrafenib (NR,NR)	BRAF inhibitor	NR	1.75	diffuse yellowish hyperkeratosis	NR	NR	keratolytic creams (NR,NR)	NR	NR	NR	NR	4
		48, M	NR	NR	metastatic melanoma	NR	dabrafenib (NR,NR)	BRAF inhibitor	NR	1.75	diffuse yellowish hyperkeratosis	NR	NR	keratolytic creams (NR,NR)	NR	NR	NR	NR	4
		57, M	NR	NR	metastatic melanoma	NR	dabrafenib (NR,NR)	BRAF inhibitor	NR	1.75	diffuse yellowish hyperkeratosis	NR	NR	keratolytic creams (NR,NR)	NR	NR	NR	NR	4
		79, M	NR	NR	metastatic melanoma	NR	dabrafenib (NR,NR)	BRAF inhibitor	NR	1.75	diffuse yellowish hyperkeratosis	NR	NR	keratolytic creams (NR,NR)	NR	NR	NR	NR	4
CS (4)[3]	2	53, M	NR	NR	stage IVa BRAF-mutant cutaneous melanoma	NR	encorafenib (300mg, daily)	BRAF inhibitor	NR	1	palmoplantar keratoderma	progressive increase of hyperkeratosis and fragmentation and loss of elastic fibers	N	NR	NR	NR	NR	NR	4
		35, M	NR	NR	stage IV BRAF-mutant cutaneous melanoma	NR	encorafenib (NR,NR)	BRAF inhibitor	NR	2	palmoplantar keratoderma	NR	N	NR	NR	NR	NR	NR	3

CS (4)[4]	1/4	57, M	NR	NR	papillary thyroid cancer	radioactive iodine (NR,NR)	vemurafenib (NR,NR)	BRAF inhibitor	NR	2	focal palmoplantar hyperkeratosis	facial papule demonstrated hyperkeratosis, acanthosis, and papillomatosis without apparent koilocytic change,	NR	NR	NR	NR	NR	NR	4
CR (5)[5]	1	70, M	NR	NR	melanoma	NR	vemurafenib 960 mg,BID)	BRAF inhibitor	NR	32	hyperkeratosis	NR	N	NR	NR	NR	NR	NR	4
CR (5)[6]	1	56, F	NR	NR	melanoma with brain metastasis	NR	vemurafenib (1920 mg,daily)	BRAF inhibitor	oral prednisolone (NR,NR)	0.5	hyperkeratosis	prominent hyperkeratosis with mild lymphocyte infiltration in the dermis	Y	NR	NR	0.5	CoR	NR	7
CS (4)[7]	1/3	50, M	NR	NR	cutaneous melanoma with distant metastases	dacarbazine monotherapy (NR,NR)	vemurafenib, 960 mg,BID)	BRAF inhibitor	NR	1	painful hyperkeratotic papules and plaques	NR	N	NR	NR	NR	PR	NR	4
CR (5)[8]	1	67, F	NR	NR	pT2a lentigo maligna melanoma	NR	vemurafenib (960mg,BID)	BRAF inhibitor	NR	NR	hyperkeratosis	NR	Y	NR	NR	1	CoR	NR	5
CR (5)[9]	1	69, F	NR	NR	hepatocellular adenocarcinoma (3 years prior)	adriamycin (NR,NR)	sorafenib(NR,NR)	Tyrosine kinase inhibitor	NR	3	mild palmar erythema and hyperkeratosis	NR	NR	topical keratolytics (NR,NR)	NR	NR	PR	NR	4
CR (5)[10]	1	47, F	NR	Hypertension	Philadelphia-chromosome positive chronic myeloid leukaemia	NR	imatinib mesylate (800mg, daily)	Tyrosine kinase inhibitor	enalapril (NR,NR)	3	erythematous and hyperkeratotic plaques	hyperkeratosis, hypergranulosis with focal areas of parakeratosis, basal layer degeneration, few apoptotic keratinocytes, band-like lympho-plasmacytic dermal infiltrate and pigment incontinence	N	oral prednisolone (0.5 mg/kg, NR)	NR	4	PR	NR	4
CR (5)[11]	1	62, M	NR	NR	gastrointestinal stromal tumors	Imatinib mesylate (NR,NR)	sunitinib (50 mg,daily, 4 weeks on, 2 weeks off with 6 weeks cycle)	Tyrosine kinase inhibitor	NR	1.5	hyperkeratosis	NR	NR	NR	NR	NR	NR	NR	4
CR (5)[12]	1	57, M	NR	NR	chronic myeloid leukaemia	NR	Imatinib mesylate (400mg,daily)	Tyrosine kinase inhibitor	NR	2	keratotic erythemas with scales	hyperkeratosis, acanthosis and band-like cellular infiltration with dilated small vessels in the upper dermis, liquefaction degeneration and	Y	antihistamine and topical corticosteroid (NR,NR)	imatinib mesylate (100mg,Daily)	6	PR	NR	8

												lymphocyte infiltration into the epidermis						
CR (5)[13]	1	55, F	NR	NR	metastatic renal cell carcinoma	NR	sorafenib (400 mg,BID)	Tyrosine kinase inhibitor	NR	1	hyperkeratosis	mild spongiosis, acanthosis and basal cell vacuolisation, upper dermis showed mild perivascular lymphomononuclear inflammatory cell infiltrate with mast cells	Y	oral corticosteroids, antihistamines, and emollients (NR,NR)	lower dose of sorafenib	0.5	CoR	N	5
CS (4)[14]	3	74, F	NR	NR	non-small cell lung cancer	gefitinib,pemetrexed, carboplatin (NR,NR)	olmutinib (800mg,NR)	Tyrosine kinase inhibitor	NR	1	asymptomatic diffuse thickening	acanthosis, hyperkeratosis, focal parakeratosis and papillary dermal elongation with minimal dermal inflammation,	Y	NR	NR	1	CoR	NR	5
		59, M	NR	NR	non-small cell lung cancer	pemetrexed and carboplatin ,erlotinib (NR,NR)	olmutinib (800mg,daily)	Tyrosine kinase inhibitor	NR	1	asymptomatic hyperkeratotic patches and plaques	NR	Y	NR	NR	1	CoR	NR	5
		54, F	NR	NR	non-small cell lung cancer	erlotinib, afatinib (NR,NR)	olmutinib (800mg, daily)	Tyrosine kinase inhibitor	NR	1	focal hyperkeratotic plaques	findings consistant with PPK	NR	NR	NR	NR	NR	NR	4
CR (5)[15]	1	73, F	NR	hypertension, presbyakusis, cataract of the left eye, embolism of his left ocular artery vertigo	cutaneous composite lymphoma	cyclophosphamide, doxorubicin, vincristine, etoposide, prednisolone (NR,NR)	pegylated liposomal encapsulated doxorubicin (NR,NR)	Chemotherapy	NR	NR	hyperkeratosis	NR	NR	topical ointment containing urea 10% (NR,NR)	NR	NR	NR	NR	4
CS (4)[16]	2	44, F	NR	NR	breast cancer	adjuvant chemotherapy (NR,NR)	oral capecitabine (1800mg, BID)	Chemotherapy	tamoxifen (NR,NR)	5	even and thick hyperkeratosis with hyperpigmentation	orthokeratotic hyperkeratosis and moderate acanthosis with hypergranulosis	NR	topical steroid cream with keratolyics (NR,NR)	NR	1	PR	NR	3
		49, F	NR	NR	breast cancer	adjuvant chemoradiotherapy (NR,NR)	oral capecitabine (2000mg, BID)	Chemotherapy	tamoxifen (NR,NR)	1	diffuse thickening and hyperpigmentation	hyperkeratosis, acanthosis and mild perivascular lymphohistiocytic infiltration in the papillary dermis.	NR	NR	NR	NR	PR	NR	5

CR (5)[17]	1	67, M	NR	NR	mycosis fungoides	NR	doxorubicin (NR,NR)	Chemotherapy	cyclophosphamide, vincristine and prednisone (NR,NR)	0.5	acute desquamative erythroderma	necrotic keratinocytes and a slight lichenoid infiltrate	Y	NR	cyclophosphamide, vincristine and prednisone	0.25	PR	NR	4
CR (5)[18]	1	68, F	NR	NR	chronic myeloid leukemia	NR	oral hydroxyurea (1.0–1.5 g,daily)	Chemotherapy	NR	12	hyperkeratosis,Bilateral, well defined, and symmetrical large shallow ulcers	epidermal atrophy with hyperkeratosis, vacuolar degeneration in basal layer, and mononuclear perivascular inflammatory infiltrate in the upper and mid-dermis	Y	NR	busulfan	6	CoR	NR	5
CR (5)[19]	1	31, F	NR	NR	sigmoidal adenocarcinoma	NR	oral tegafur (400 mg, BID)	Chemotherapy	NR	10	well demarcated erythema, hyperkeratosis with scaling and fissuring	hyperkeratosis, a few scattered necrotic keratinocytes, and mild vacuolar degeneration of the basal cell layer with a mild perivascular lymphohistiocytic infiltrate in the papillar dermis	Y	topical steroids and 6% salicylic acid and vitamin B6 (100 mg/d orally).	NR	5	CoR	NR	5
CR (5)[20]	1	85, M	NR	Chronic heart failure	T-cell leukaemia/lymphoma	NR	mogamulizumab (NR,NR)	Biologic, anti-CCR4 antibody	spironolactone, tamsulosin, bisoprolol fumarate, carbocisteine and potassium chloride (NR,NR)	3	hyperkeratosis	hyperkeratosis with exocytosis of small lymphocytes without any atypia, vacuolar alteration in the junctional zone and marked perivascular lymphocytic infiltrates in the dermis	Y	prednisolone (30mg,daily)	NR	NR	PR	N	4
CR (5)[21]	1	49, M	NR	NR	melanoma	NR	pembrolizumab (NR,NR)	Biologic, anti-PD1 antibody	Ipilimumab (NR,NR)	NR	hyperkeratosis	NR	NR	NR	NR	NR	NR	NR	3
CR (5)[22]	1	51, F	NR	NR	Sezary syndrome	CHOP regimen (NR,NR)	bexarotene (450 mg,daily)	Retinoid X receptor inhibitor	NR	0.25	palmoplantar hyperkeratosis	NR	Y	NR	NR	NR	CoR	NR	5
CR (5)[23]	1	55, M	NR	NR	cutaneous T-cell lymphoma	topical corticosteroid cream, psoralen, UVA phototherapy and interferon α	bexarotene (300 mg,daily)	Retinoid X receptor inhibitor	NR	48	palmoplantar keratoderma	NR	N	NR	bexarotene and denileukin diftitox	NR	PR	NR	4
CR (5)[24]	1	51, M	NR	NR	NR	fosinopril (NR,NR)	metoprolol/hydrochlorothiazide (NR,NR)	Beta blocker, diuretic	NR	6	keratoderma with painful fissuring	superficial dermal infiltrate composed of numerous atypical lymphocytes with convoluted and cerebriform nuclei	Y	NR	NR	NR	CoR	NR	4

												Prominent epidermotropism was present							
CR (5)[25]	1	85, M	NR	NR	NR	NR	lisinopril (NR,NR)	ACEi	levothyroxine, aspirin, allopurinol, atenolol, doxepin and vitamin B12 (NR,NR)	4	bilateral palmoplantar keratoderma with painful sores	NR	Y	NR	NR	0.75	PR	NR	4
CR (5)[26]	1	55, F	NR	NR	hypertension	NR	losartan (NRNR)	ARB	NR	12	symmetric palmoplantar hyperkeratoris with hemorrhagic fissures	marked epidermal hyperplasia with significant hypergranulosis hyperkeratosis, slight spongiosis and necrotic keratinocytes	Y	topical and systemic corticosteroids, vitamin D analogs, urea, and other keratolytics	NR	1	CoR	NR	4
CS (4)[27]	2 (1)	69, M	NR	Wegener granulomatosis	Sezary syndrome	NR	cyclosporine (NR,NR)	Calcineurin inhibitor	mycophenolate, and prednisone (NR,NR)	90	scaling with painful fissures and hyperkeratosis	NR	Y	NR	oral bexarotene and topical nitrogen mustard	NR	CoR	NR	4
CR (5)[28]	1	49, F	NR	NR	HIV	NR	trizivir,efavirenz (NR,NR)	Antiretrovirals	tenofovir and emtricitabin (NR,NR)	3	hyperkeratosis	Compact orthokeratotic hyperkeratosis, acanthosis and hypergranulosis	Y	low-dose acitretin (10–20 mg, daily)	NR	NR	PR	NR	8
CR (5)[29]	1	50, F	NR	frequent migraines	Season Flu vaccine	influenza vaccination	influenza vaccination (NR,NR)	Vaccine	NR	0.25	thickened with desquamation and painful fissuring.	NR	NR	acitretin (10 mg daily)	NR	6	PR	NR	7
CR (5)[30]	1	11, F	NR	NR	epilepsy	sodium valproate (NR,NR)	Ayurvedic medications (NR,NR)	Naturopathic	NR	6	multiple, discrete, keratotic papules	marked lamellar hyperkeratosis and a sparse perivascular infiltrate of lymphocytes and histiocytes in the dermis	Y	topical keratolytic agent	NR	6	CoR	NR	8

Cohort Studies and Randomized Controlled Trials

Study design (Level of Evidence)	Drug	Drug Class	Sample size (n/N)	Indication	Inclusion Criteria	Exclusion Criteria	Percentage of patients with PPK (%)	Mean Latency Period (months)	Mean age of patients who developed PPK	Sex of patients who developed PPK (F:M)	PPK Severity (ADR Grade)	PPK treatment	Naranj ADR Scale
CH(4)[31]	Dabrafenib (150mg,BID)	BRAF inhibitor	8/40	multiple myeloma	Multiple myeloma patients over the age of 18 with BRAF V600 mutation	pregnancy, prior therapy with BRAF inhibitors, and known or suspected hypersensitivity to any component of Dabrafenib	20.0	NR	NR	NR	1-2	curettage and topic keratolytic	4

CH(4)[32]	Vemurafenib (960mg,BID)	BRAF inhibitor	2/43	multiple myeloma	Metastatic unresectable stage IIIC or IV multiple myeloma patients over the age of 18 with BRAF mutations	NR	4.7	NR	NR	NR	1-2	NR	4
RCT(1b)[33]	Encorafenib (450mg,daily) and binimetinib (45mg,BID)	BRAF inhibitor and MEK1/2 inhibitor	18/192	melanoma	Locally advanced, unresectable, or metastatic cutaneous melanoma or unknown primary melanoma stage IIIB, IIIC, or IV in patients over the age of 18. Patients were treatment naive or had progressed on or after previous first-line immunotherapy and had a BRAFV600E or BRAFV600K mutation	Previous or concurrent malignancy with exceptions, prior therapy with a BRAF inhibitor and/or a MEK inhibitor; previous systemic chemotherapy treatment, extensive radiotherapy or investigational agent other than immunotherapy, or more than one line of immunotherapy for locally advanced unresectable or metastatic melanoma	9.4	NR	NR	NR	grade 1-2: 18/18	NR	4
	Encorafenib (300mg,daily)	BRAF inhibitor	50/192				26.0	NR	NR	NR	grade 1-2: 47/50; grade 3: 3/50	NR	
	Vemurafenib (960mg,BID)	BRAF inhibitor	31/186				16.7	NR	NR	NR	grade 1-2: 29/31; grade 3: 2/31	NR	
CH(4)[34]	Vemurafenib or dabrafenib monotherapy	BRAF inhibitor	16/47	melanoma	Patients treated with vemurafenib, dabrafenib, or dabrafenib and trametinib	patients presenting new melanocytic tumours or changes in their pre-existing naevi were	34.0	NR	54.5	6:14	NR	NR	4
	Dabrafenib and trametinib	BRAF inhibitor and MEK1/2 inhibitor	4/12				33.3	NR			NR	NR	
CH(4)[35]	Vemurafenib (960mg,daily) or dabrafenib monotherapy (300mg,daily)	BRAF inhibitor	NR/49	melanoma	BRAFi monotherapy (vemurafenib or dabrafenib) for metastatic Stage IV or unresectable Stage III melanoma	NR	NR	NR	NR	NR	NR	NR	4
CS(4)[36]	Binimetinib	MEK1/2 inhibitor	1/25	melanoma	Patients with BRAF V600- or NRAS-mutated melanoma treated with BRAFi and/or MEKi (vemurafenib, dabrafenib, encorafenib, trametinib, binimetinib) within approved clinical trials	Patients who withdrew consent and those whom unblinding was not possible	4.0	NR	NR	NR	NR	NR	4
	Encorafenib	BRAF inhibitor	13/24				54.2	NR	NR	NR	NR	NR	
	Vemurafenib	BRAF inhibitor	3/6				50.0	NR	NR	NR	NR	NR	
	Encorafenib and binimetinib	BRAF inhibitor and MEK1/2 inhibitor	5/49				10.2	NR	NR	NR	NR	NR	
RCT (2b)[37]	dabrafenib (150mg, BID) and trametinib (2mg, daily)	BRAF inhibitor and MEK1/2 inhibitor	11/209	melanoma	previously untreated patients with BRAF V600E/K-mutant unresectable stage IIIC or stage IV melanoma	NR	5.3	NR	NR	NR	grade 1-2: 10/209; grade 3: 1/209	NR	4
	dabrafenib (150mg, BID) and placebo	BRAF inhibitor	39/211				18.5		NR	NR	grade 1-2: 38/209; grade 3: 1/209	NR	
CS(4)[38]	Vemurafenib (960mg,BID)	BRAF inhibitor	4/28	melanoma	Patients with metastatic melanoma	NR	14.3	NR	NR	NR	NR	NR	4

CH (2b)[39]	Vemurafenib (960mg,BID)	BRAF inhibitor	3/26	hairy cell leukemia	Harry cell leukemia patients over the age of 18 with BRAF-V600E mutations refractory to chemotherapy and/or immunotherapy	Prior bone marrow transplant, concurrent anti-cancer therapy, pregnancy, known hypersensitivity to BRAF inhibitors	11.5	NR	NR	NR	2	NR	4
CH (4)[40]	tofogliflozin (20mg,BID)	SGLT2 inhibitor	1/6712	type 2 diabetes mellitus	Tofogliflozin-naïve patients with type 2 diabetes mellitus without limitation in age, HbA1c, concomitant medications, and disease history	NR	0.01	NR	NR	NR	NR	NR	3

Abbreviations: ACEi angiotensin converting enzyme inhibitor; ARB angiotensin II receptor blocker; CH cohort study; CHOP cyclophosphamide, doxorubicin, vincristine, and prednisone; CoR complete remission; CR case report; CS case series; F female; M male; MEK1/2 mitogen-activated protein kinase kinase 1/2; NoR no resolution; NR not reported; PR partial resoltuion; RCT randomized controlled trial

References

1. Mourah S, Lorillon G, Meignin V, et al. Dramatic transient improvement of metastatic BRAFV600E-mutated Langerhans cell sarcoma under treatment with dabrafenib. *Blood*. 2015;126(24):2649-2652. doi:http://dx.doi.org/10.1182/blood-2015-06-650036
2. Lacroix JP, Wang B. Prospective case series of cutaneous adverse effects associated with dabrafenib and trametinib. *Journal of Cutaneous Medicine and Surgery*. 2017;21(1):54-59. doi:http://dx.doi.org/10.1177/1203475416670368
3. Fernandez-Sartorio C, Boada A, Chavez-Bourgeois MM, et al. Aged-looking skin and encorafenib: An adverse event of BRAF inhibitors. *Melanoma Research*. 2018;28(2):160-162. doi:http://dx.doi.org/10.1097/CMR.0000000000000422
4. Chu EY, Wanat KA, Miller CJ, et al. Diverse cutaneous side effects associated with BRAF inhibitor therapy: A clinicopathologic study. *Journal of the American Academy of Dermatology*. 2012;67(6):1265-1272. doi:http://dx.doi.org/10.1016/j.jaad.2012.04.008
5. Lloyd-Lavery A, Hodgson T, Coupe N, et al. Delayed oral toxicity from long-term vemurafenib therapy. *British Journal of Dermatology*. 2016;174(5):1159-1160. doi:http://dx.doi.org/10.1111/bjd.14457
6. Furudate S, Fujimura T, Kambayashi Y, et al. Keratoacanthoma, palmoplantar keratoderma developing in an advanced melanoma patient treated with vemurafenib regressed by blockade of mitogen-activated protein kinase kinase signaling. *Journal of Dermatology*. 2017;44(9):e226-e227. doi:http://dx.doi.org/10.1111/1346-8138.13898
7. Huang V, Hepper D, Anadkat M, Cornelius L. Cutaneous toxic effects associated with vemurafenib and inhibition of the BRAF pathway. *Archives of Dermatology*. 2012;148(5):628-633. doi:http://dx.doi.org/10.1001/archdermatol.2012.125
8. Lee SB, Weide B, Ugurel S, Mossner R, Enk A, Hassel JC. Vemurafenib-induced granuloma annulare. *JDDG - Journal of the German Society of Dermatology*. 2016;14(3):305-308. doi:http://dx.doi.org/10.1111/ddg.12777
9. Alli N, Dogan S, Tantoglu BH. Palmoplantar keratoderma like drug eruption due to sorafenib. *Turk Dermatoloji Dergisi*. 2018;12(1):50-51. doi:http://dx.doi.org/10.4274/tdd.2395
10. Arshdeep, De D, Malhotra P, Saikia U. Imatinib mesylate-induced severe lichenoid rash. *Indian Journal of Dermatology, Venereology and Leprology*. 2014;80(1):93-95. doi:http://dx.doi.org/10.4103/0378-6323.125505
11. Demirci U, Coskun U, Erdem O, et al. Acne rosacea associated imatinib mesylate in a gastrointestinal stromal tumor patient. *Journal of Oncology Pharmacy Practice*. 2011;17(3):285-287. doi:http://dx.doi.org/10.1177/1078155210374674
12. Kuraishi N, Nagai Y, Hasegawa M, Ishikawa O. Lichenoid drug eruption with palmoplantar hyperkeratosis due to imatinib mesylate: A case report and a review of the literature. *Acta Dermato-Venereologica*. 2010;90(1):73-76. doi:http://dx.doi.org/10.2340/00015555-0758
13. Guliani A, Daroach M, Aggarwal D, Radotra BD, Kumaran MS. Severe hand-foot skin reaction and erythema multiforme-like lesions due to sorafenib. *Postgraduate Medical Journal*. 2018;94(1115):535-536. doi:http://dx.doi.org/10.1136/postgradmedj-2018-136027
14. Chen KL, Cho YT, Yang CW, et al. Olmutinib-induced palmoplantar keratoderma. *British Journal of Dermatology*. 2018;178(2):e129-e131. doi:http://dx.doi.org/10.1111/bjd.15935
15. Wollina U, Langner D, Hansel G, Haroske G. Pegylated liposomal-encapsulated doxorubicin in cutaneous composite lymphoma: A case report. *Medicine (United States)*. 2016;95(43):e4796. doi:http://dx.doi.org/10.1097/MD.0000000000004796
16. Do JE, Kim YC. Capecitabine-induced diffuse palmoplantar keratoderma: Is it a sequential event of hand-foot syndrome? *Clinical and Experimental Dermatology*. 2007;32(5):519-521. doi:http://dx.doi.org/10.1111/j.1365-2230.2007.02451.x
17. Lamoureux C, Gerard E, Ouhabrache N, Toukal F, Pham-Ledard A, Beylot-Barry M. A case of potential radiation recall dermatitis induced by chemotherapy, following low-dose total-skin electron beam therapy. *European Journal of Dermatology*. 2019;29(6):671-672. doi:http://dx.doi.org/10.1684/ejd.2019.3670
18. Nofal A, El-Din ES. Hydroxyurea-induced dermatomyositis: True amyopathic dermatomyositis or dermatomyositis-like eruption? *International Journal of Dermatology*. 2012;51(5):535-541. doi:http://dx.doi.org/10.1111/j.1365-4632.2011.05105.x
19. Jucgla A, Sais G, Navarro M, Peyri J. Palmoplantar keratoderma secondary to chronic acral erythema due to tegafur. *Archives of Dermatology*. 1995;131(3):364-365. doi:http://dx.doi.org/10.1001/archderm.131.3.364

20. Ito A, Sugita K, Adachi K, Hosoda Y, Motokura T, Yamamoto O. CD8+ T-cell-mediated interface dermatitis after CCR4+ T-cell depletion by mogamulizumab treatment of adult T-cell leukaemia/lymphoma. *Acta Dermato-Venereologica*. 2017;97(3):377-378. doi:http://dx.doi.org/10.2340/00015555-2555
21. Kirchberger MC, Hauschild A, Schuler G, Heinzerling L. Combined low-dose ipilimumab and pembrolizumab after sequential ipilimumab and pembrolizumab failure in advanced melanoma. *European Journal of Cancer*. 2016;65((Kirchberger, Schuler, Heinzerling) Department of Dermatology, University Hospital Erlangen, Erlangen, Germany(Hauschild) Department of Dermatology, University Hospital Schleswig-Holstein, Kiel, Germany):182-184. doi:http://dx.doi.org/10.1016/j.ejca.2016.07.003
22. Bagazgoitia L, Perez-carmona L, Rios L, Munoz E, Harto A, Jaen P. Acute hyperkeratotic and desquamative reaction in a patient with Sezary syndrome treated with bexarotene [19]. *Journal of the European Academy of Dermatology and Venereology*. 2008;22(3):389-390. doi:http://dx.doi.org/10.1111/j.1468-3083.2007.02341.x
23. Cervigon-Gonzalez I, Torres-Iglesias LM, Palomo-Arellano A, Gil-Pascual B. Advanced-stage primary cutaneous T-cell lymphoma treated with bexarotene and denileukin diftitox. *Case Reports in Dermatology*. 2011;3(1):13-17. doi:http://dx.doi.org/10.1159/000324185
24. Reeder MJ, Wood GS. Drug-induced pseudo-sezary syndrome: A case report and literature review. *American Journal of Dermatopathology*. 2015;37(1):83-86. doi:http://dx.doi.org/10.1097/DAD.0000000000000169
25. Schmutz JL, Barbaud A, Trechot P. Lisinopril-induced erythroderma. *Annales de Dermatologie et de Venereologie*. 2009;136(5):486. doi:http://dx.doi.org/10.1016/j.annder.2009.02.001
26. Calvo M, Fernández-Guarino M, Martín-Saez E, Carrillo R, Garate M. [Palmoplantar hyperkeratosis associated with losartan]. *Actas Dermosifiliogr*. Sep 2006;97(7):463-6. doi:10.1016/s0001-7310(06)73442-1
27. Pomerantz RG, Campbell LS, Jukic DM, Geskin LJ. Posttransplant cutaneous T-cell lymphoma: Case reports and review of the association of calcineurin inhibitor use with posttransplant lymphoproliferative disease risk. *Archives of Dermatology*. 2010;146(5):513-516. doi:http://dx.doi.org/10.1001/archdermatol.2010.60
28. Verdolini R, Clayton N, Arkoumani E. Striate palmar keratoderma and antiretroviral treatment for human immunodeficiency virus infection: Not just a coincidence. *Clinical and Experimental Dermatology*. 2013;38(5):556-558. doi:http://dx.doi.org/10.1111/ced.12005
29. Lim D, Rademaker M, Gardner D, Oakley A. Palmoplantar keratoderma: An adverse reaction to influenza vaccination. *Australasian Journal of Dermatology*. 2011;52(4):298-300. doi:http://dx.doi.org/10.1111/j.1440-0960.2011.00817.x
30. Khandpur S, Malhotra AK, Bhatia V, et al. Chronic arsenic toxicity from Ayurvedic medicines. *International Journal of Dermatology*. 2008;47(6):618-621. doi:http://dx.doi.org/10.1111/j.1365-4632.2008.03475.x
31. Cocorocchio E, Gandini S, Alfieri S, et al. Dabrafenib in metastatic melanoma: A monocentric 'real life' experience. *ecancermedicalscience*. 2016;10((Cocorocchio, Alfieri, Battaglia, Di Leo, Riviello, Pala, Martinoli, Ferrucci) Medical Oncology of Melanoma and Sarcoma Division, Istituto Europeo di Oncologia, via Ripamonti 435, Milan 2014, Italy(Gandini) Biostatistics Division, Istituto Europeo di Onco):624. doi:http://dx.doi.org/10.3332/ecancer.2016.624
32. Czirbesz K, Gorka E, Balatoni T, et al. Efficacy of Vemurafenib Treatment in 43 Metastatic Melanoma Patients with BRAF Mutation. Single-Institute Retrospective Analysis, Early Real-Life Survival Data. *Pathology and Oncology Research*. 2019;25(1):45-50. doi:http://dx.doi.org/10.1007/s12253-017-0324-1
33. Dummer R, Ascierto PA, Gogas HJ, et al. Encorafenib plus binimetinib versus vemurafenib or encorafenib in patients with BRAF-mutant melanoma (COLUMBUS): a multicentre, open-label, randomised phase 3 trial. *The Lancet Oncology*. 2018;19(5):603-615. doi:http://dx.doi.org/10.1016/S1470-2045%2818%2930142-6
34. Erfan G, Puig S, Carrera C, et al. Development of cutaneous toxicities during selective anti-BRAF therapies: Preventive role of combination with MEK inhibitors. *Acta Dermato-Venereologica*. 2017;97(2):258-260. doi:http://dx.doi.org/10.2340/00015555-2488
35. Finon A, Zaragoza J, Maillard H, et al. A high neutrophil to lymphocyte ratio prior to BRAF inhibitor treatment is a predictor of poor progression-free survival in patients with metastatic melanoma. *European Journal of Dermatology*. 2018;28(1):38-43. doi:http://dx.doi.org/10.1684/ejd.2017.3167
36. Graf NP, Koelblinger P, Galliker N, et al. The spectrum of cutaneous adverse events during encorafenib and binimetinib treatment in B-rapidly accelerated fibrosarcoma-mutated advanced melanoma. *Journal of the European Academy of Dermatology and Venereology*. 2019;33(4):686-692. doi:http://dx.doi.org/10.1111/jdv.15363
37. Long GV, Flaherty KT, Stroyakovskiy D, et al. Dabrafenib plus trametinib versus dabrafenib monotherapy in patients with metastatic BRAF V600E/ K-mutant melanoma: Long-term survival and safety analysis of a phase 3 study. *Annals of Oncology*. 2017;28(7):1631-1639. doi:http://dx.doi.org/10.1093/annonc/mdx176
38. Rinderknecht JD, Goldinger SM, Rozati S, et al. RASopathic Skin Eruptions during Vemurafenib Therapy. *PLoS ONE*. 2013;8(3):e58721. doi:http://dx.doi.org/10.1371/journal.pone.0058721
39. Tiacci E, Park JH, De Carolis L, et al. Targeting mutant BRAF in relapsed or refractory hairy-cell leukemia. *New England Journal of Medicine*. 2015;373(18):1733-1747. doi:http://dx.doi.org/10.1056/NEJMoa1506583
40. Utsunomiya K, Kakiuchi S, Senda M, et al. Safety and effectiveness of tofogliflozin in Japanese patients with type 2 diabetes mellitus: Results of 24-month interim analysis of a long-term post-marketing study (J-STEP/LT). *Journal of Diabetes Investigation*. 2020;11(4):906-916. doi:http://dx.doi.org/10.1111/jdi.13233

Table S3. Summary of case reports of palmoplantar keratoderma as an adverse drug reaction

Drug Class	Drug Name (n)	Indication	Mean Latency Period (Range) (n)	Drug Discontinuation	Resolution	Treatment	Mean Resolution Period	Medications prescribed for original indication	Recurrence of PPK	Naranjo score (Interpretation)
**BRAF Inhibitors (n=13)**	encorafenib (n=2)<br>dabrafenib (n=6)<br>vemurafenib (n=5)	Melanoma (n=11)<br>Langerhans cell histiocytosis (n=1)<br>Papillary thyroid cancer (n=1)	4.2 months (0.5-32) (n=12)<br>NR (n=1)	Y (n=3)<br>N (n=4)<br>NR (n=6)	CoR (n=2)<br>---------------<br>PR (n=1)	drug discontinued (n = 2)<br>--------------------------------------<br>drug discontinued (n=1)	0.75 months (0.5-1) (n=2)<br>NR (n=1)	NR (n=3)	NR (n=3)	4<br>(Possible)
**Tyrosine kinase inhibitors (n=8)**	olmutinib (n=3)<br>imatinib (n=2)<br>sorafenib (n=2)<br>sunitinib (n=1)	Chronic myeloid leukemia (n=2)<br>Non-small cell lung cancer (n=3)<br>Renal cell carcinoma (n=1)<br>Hepatocellular adenocarcinoma (n=1)<br>Gastrointestinal stromal tumor (n=1)	1.7 months (1-3) (n=8)	Y (n=4)<br>N (n=1)<br>NR (n=3)	CoR (n=3)<br>---------------<br>PR (n=3)	oral corticosteroids and antihistamines (n=1)<br>drug discontinued (n =3)<br>--------------------------------------<br>oral corticosteroids (n=1)<br>topical keratolytic (n=1)<br>topical corticosteroids and antihistamines<br>drug discontinued (n=1)	2.5 months (0.5-6) (n=5)<br>NR (n=1)	Lower dose of imatinib (n=1)<br>Lower dose of sorafenib (n=1)<br>NR (n=2)	N (n=1)<br>NR (n=7)	5<br>(Probable)
**Chemotherapy (n=6)**	doxorubicin (n=2)<br>capecitabine (n=2)<br>hydroxyurea (n=1)<br>tegafur (n=1)	Breast cancer (n=2)<br>Cutaneous composite lymphoma (n=1)<br>Mycosis fungoides (n=1)<br>Chronic myeloid leukemia (n=1)<br>Sigmoid adenocarcinoma (n=1)	5.7 months (0.5-12) (n=5)	Y (n=3)<br>NR (n=3)	CoR (n=2)<br>---------------<br>PR (n=3)	topical corticosteroids and keratolytic (n=1)<br>drug discontinued (n =2)<br>--------------------------------------<br>topical corticosteroids and keratolytic (n=1)<br>drug discontinued (n=1)	3.1 months (0.25-6) (n=4)<br>NR (n=2)	cyclophosphamide, vincristine and prednisone (n=1)<br>busulfan (n=1)<br>NR (n=3)	NR (n=5)	5<br>(Probable)
**Antihypertensive Medications (n=3)**	Metoprolol and hydrochlorothiazide (n=1)<br>Lisinopril (n=1)<br>Losartan (n=1)	Hypertension (n=1)<br>NR (n=2)	7.3 months (4-12) (n=3)	Y (n=3)	CoR (n=2)<br>---------------<br>PR (n=1)	topical and systemic corticosteroids, vitamin D analogs, and keratolytics (n=1)<br>drug discontinued (n=1)<br>--------------------------------------<br>drug discontinued (n=1)	0.9 months (0.75-1) (n=2)<br>NR (n=1)	NR (n=3)	NR (n=3)	4<br>(Possible)
**Biologics (n=2)**	mogamulizumab (n=1)<br>pembrolizumab (n=1)	Melanoma (n=1)<br>Adult T-cell leukaemia/lymphoma (n=1)	3 months (n=1)	Y (n=1)<br>NR (n=1)	PR (n=1)	oral corticosteroids (n=1)<br>drug discontinued (n =1)	NR (n=1)	NR (n=1)	N (n=1)	4<br>(Possible)
**Retinoids (n=2)**	Bexarotene (n=2)	Sezary syndrome (n=1)<br>Cutaneous T-cell lymphoma (n=1)	24.1 months (0.25-48) (n=2)	Y (n=1)<br>N (n=1)	CoR (n=1)<br>---------------<br>PR (n=1)	drug discontinued (n=1)<br>--------------------------------------<br>NR (n=1)	NR (n=2)	NR (n=2)	NR (n=2)	5<br>(Probable)
**Immunosuppressives (n=1)**	Cyclosporine (n=1)	Sezary syndrome (n=1)	90 months (n=1)	Y (n=1)	CoR (n=1)	drug discontinued (n=1)	NR (n=1)	oral bexarotene and topical nitrogen mustard (n=1)	NR (n=1)	4<br>(Possible)
**Antiretrovirals (n=1)**	Trizivir, efavirenz (n=1)	HIV (n=1)	3 months (n=1)	Y (n=1)	PR (n=1)	keratolytic (n=1)<br>drug discontinued (n=1)	NR (n=1)	NR (n=1)	NR (n=1)	8<br>(Probable)

**Vaccination (n=1)**	Influenza vaccine (n=1)	Annual immunization (n=1)	0.25 months (n=1)	NR (n=1)	PR (n=1)	keratolytic (n=1)	6 months (n=1)	NR (n=1)	NR (n=1)	7 (Probable)
**Naturopathic (n=1)**	Ayurvedic medications (n=1)	Epilepsy (n=1)	6 months (n=1)	Y (n=1)	CoR (n=1)	keratolytic (n=1)<br>drug discontinued (n=1)	6 months (n=1)	NR (n=1)	NR (n=1)	8 (Probable)

Abbreviations: CoR complete remission; F female; HIV human immunodeficiency virus; M male; N no; NR not reported; PR partial resolution; Y yes.
